# Preparation and Characterization of Low-Molecular-Weight Polyacrylonitrile

**DOI:** 10.3390/polym17081112

**Published:** 2025-04-19

**Authors:** Yuanteng Yang, Xiaoli Jiang, Jing Jiang, Yang Liu, Lin Zhao, Hongyu Zhu, Junjie Wang, Zongkai Yan, Yagang Zhang

**Affiliations:** 1 School of Materials and Energy, University of Electronic Science and Technology of China, Chengdu 611731, China; 202221030325@std.uestc.edu.cn (Y.Y.); jiangxl@std.uestc.edu.cn (X.J.); 202321030310@std.uestc.edu.cn (J.J.); 202221030136@std.uestc.edu.cn (Y.L.); zhaolin316@uestc.edu.cn (L.Z.); zhy@uestc.edu.cn (H.Z.); 2 Department of Chemistry, School of Science, Xihua University, Chengdu 610039, China

**Keywords:** polyacrylonitrile, low molecular weight, thermal stability, aqueous precipitation polymerization, chain transfer

## Abstract

Polyacrylonitrile (PAN) is renowned for its excellent physical and chemical properties, making it a promising candidate for producing high-performance and energetic materials. However, traditional high-molecular-weight PAN suffers from poor solubility and low reactivity, which limits its application as a precursor for advanced materials. To overcome these issues, this study successfully synthesized low-molecular-weight PAN (M_η_: 6.808 kDa) using an environmentally friendly aqueous precipitation polymerization method, utilizing ammonium persulfate (6 wt% relative to the monomer mass) as the initiator and isopropanol (400 wt%) as the chain transfer agent. The structures and properties of the synthesized low-molecular-weight PAN were analyzed in depth. The morphology and chain structure of PAN were characterized using field-emission scanning electron microscopy (FE-SEM), Fourier-transform infrared spectroscopy (FT-IR), and nuclear magnetic resonance hydrogen spectroscopy (^1^H NMR). The thermal properties were assessed using thermogravimetric analysis (TGA) and differential scanning calorimetry (DSC). Additionally, the state changes during the heating process of PAN with different molecular weights were directly observed using a visual melting point analyzer for the first time. Furthermore, the influence of molecular weight on PAN’s solubility was investigated in detail. Based on that, a linear regression between the viscosity average molecular weight (M_η_) and the number average molecular weight (M_n_) was established, providing simple and rapid access to the molecular weight of the synthesized PAN via viscosity measurements. Our study employed CTA-controlled aqueous precipitation polymerization to prepare low-molecular-weight PAN, which possesses significant potential in producing tetrazole-based energetic materials.

## 1. Introduction

Initially studied in the 1930s, polyacrylonitrile (PAN) was one of the most important and widely used synthetic polymers that served as a versatile precursor in many industries, particularly in the production of high-performance materials, including PAN fibers, carbon fibers, thermal insulation materials, etc. [1]. After decades of development and research, PAN has found significant applications in aerospace, the automotive industry, and sports equipment manufacturing [2,3].

**Figure 1:**
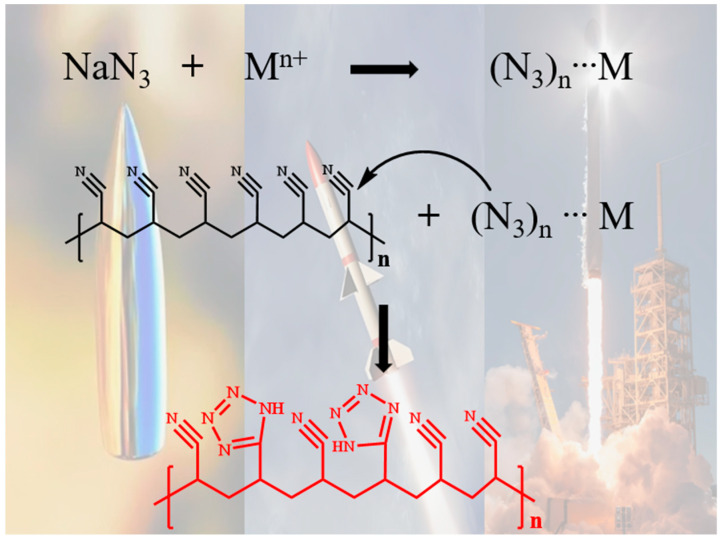
Mechanism of in situ synthesis of tetrazole from PAN and azides.

Recently, the advancement of energetic materials has sparked the resurgence of PAN research, particularly in the study of low-molecular-weight PAN [4,5]. Low-molecular-weight PAN was found to be an ideal precursor for the synthesis of energetic materials, such as nitrogen-rich metal–organic frameworks and tetrazole compounds [6]. For example, the [3 + 2] cyclization reaction between PAN and metal azides can generate tetrazole-functionalized PAN (Figure 1), which can be utilized in explosives or propellants [7,8]. In the cyclization process, the dispersion of PAN particles significantly impacts the reaction efficiency [9]. Low-molecular-weight PAN offers better dispersion, thus facilitating more complete reactions with azide ions and enhancing the conversion rate [10]. However, low-molecular-weight PAN is not readily commercially available, and research on its synthesis methods has attracted increasing attention [11].

PAN is primarily produced using solution polymerization, suspension polymerization, and bulk polymerization methods [12]. However, producing low-molecular-weight PAN using these traditional methods presents several challenges, particularly in controlling both the molecular weight and the molecular weight distribution during the polymerization process [13]. Solving these issues often necessitates sophisticated reaction designs and precise control over conditions. Additionally, complex purification and post-treatment processes are required to remove by-products and unreacted monomers, ensuring the quality and consistency of the obtained low-molecular-weight PAN [14,15]. Our team has previously employed the molecular recognition and assembly approach to precisely synthesize various functionally oriented and well-defined polymers [16,17]. Polymers with high fidelity and recognition specificity could be achieved via molecular recognition and imprinting [18]. However, the synthesis of low-molecular-weight polymer precursors for energetic materials is challenging. Developing simple and green methods for the controllable synthesis of low-molecular-weight PAN is highly desired and urgent in this field [19].

Aqueous precipitation polymerization is a widely used green and cost-effective method for synthesizing polymers, making it an ideal candidate for preparing low-molecular-weight PAN [20,21]. Based on the type of initiator, aqueous precipitation polymerization can be categorized into thermal initiation and redox initiation [22,23]. Compared with redox initiation, thermal initiation using persulfate as an initiator offers several advantages, including easier control of conditions, simpler operation and purification, and lower costs [24]. However, using aqueous precipitation polymerization with thermal initiation to synthesize low-molecular-weight PAN still presents challenges in controlling the molecular weight and weight distribution [25]. For instance, Zhao et al. employed dimethyl sulfoxide and water as a mixed solvent with ammonium persulfate as the initiator, but the resulting polyacrylonitrile sample only reached a reduced M_w_ of 200 kDa [26].

One of the most effective strategies to address this issue is to restrict the excessive growth of polymer chains via chain transfer mechanisms, where a chain transfer agent (CTA), such as alcohol, plays a crucial role in the polymerization process [27]. Monteiro et al. investigated the role of isopropanol via emulsion polymerization for synthesizing low-molecular-weight poly(vinylidene fluoride) while confirming that a CTA reduces reaction rates. However, emulsion polymerization faces challenges, such as environmental concerns from solvents and complex post-treatment processes [28]. Çetinkaya et al. explored the effect of isopropanol on the aqueous precipitation polymerization of methyl methacrylate. The authors synthesized poly(methyl methacrylate) with an M_w_ of 300 kDa by adding 1.2 wt.% isopropanol, demonstrating the efficacy of isopropanol in controlling aqueous precipitation polymerization. Nevertheless, the authors did not systematically investigate the influence of various polymerization conditions on molecular weight regulation [29].

**Figure 2:**
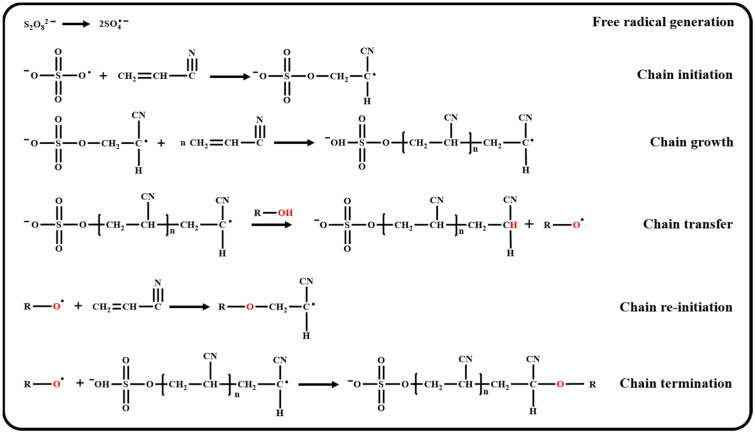
Mechanism of CTA regulating molecular weight of PAN.

The detailed mechanism of the CTA technique is illustrated in Figure 2. Upon reaching the decomposition temperature, persulfate ions generate free radicals that initiate the polymerization of acrylonitrile (chain initiation) [30]. During the propagation process (chain growth), a chain transfer reaction occurs when the hydrogen atoms are transferred from CTAs (e.g., ROH) to the active chain end, forming a new active center, RO• (chain transfer) [31]. While some RO• radicals propagate new polymer chains (chain re-initiation), the majority of RO• radicals contribute to the termination of the reaction (chain termination) [32]. Therefore, introducing a CTA can effectively control the molecular weight of polymers by limiting the growth of polymer chains and redistributing the active centers. Previous studies have shown that longer-chain alcohols enhance chain transfer activity, enabling precise control over molecular weight by prematurely terminating polymer growth. However, this benefit comes at the cost of reduced yield, as fewer active chains reach high molecular weights [33]. Although using alcohols as CTAs might negatively affect the conversion yield, carefully controlling the alcohol concentration may minimize its impact on the yield and produce the desired low-molecular-weight polymers [34,35].

Herein, low-molecular-weight PAN was synthesized via aqueous precipitation polymerization. Molecular weight regulation was achieved via strategic alcohol incorporation with a CTA. Moreover, a systematic investigation was performed to elucidate the chain length dependence of the CTA and its addition amounts on molecular weight regulation. Furthermore, the impacts of the initiator concentration and polymerization time on the conversion yield and molecular weight were studied in depth. The synthesized PAN was characterized using field-emission scanning electron microscopy (FE-SEM), Fourier-transform infrared spectroscopy (FT-IR), and nuclear magnetic resonance hydrogen spectroscopy (^1^H NMR). In addition, the thermal properties were investigated using thermogravimetric analysis (TGA) and differential scanning calorimetry (DSC). Direct observation of the color/state changes of PAN at elevated temperatures was also documented. Moreover, the solubilities of PAN with different molecular weights in various solvents and at different concentrations were comprehensively compared. Based on that, the average molecular weight of PAN was measured using the viscosity method (M_η_) and gel permeation chromatography (GPC; M_n_). A linear relationship between M_n_ and M_η_ was established, which might provide a simple and rapid characterization and evaluation for the synthesized low-molecular-weight polymer.

## 2. Materials and Methods

### 2.1. Materials

Acrylonitrile (99%) was purchased from Sigma-Aldrich (Shanghai, China) and distilled using a rotary evaporator to remove polymerization inhibitors. Deionized water is obtained through the WP-R0-10B pure water machine (Chengdu, China). Ammonium persulfate (APS; 98%), isopropanol (IPA; >99%), acetone (99%), ethylene glycol (99%), 1,4-butanediol (99.9%), N, N-dimethylformamide (DMF; 99.5%), and tetrahydrofuran (99%) were purchased from Sigma-Aldrich (Shanghai, China) and used directly. Commercially available polyacrylonitrile (PAN-4; 98%) was purchased from Meryer (Shanghai, China) for comparison.

### 2.2. Synthesis of Low-Molecular-Weight PAN

An amount of 100 mL of deionized water was added to a 250 mL three-necked flask and heated to 45 °C. Nitrogen (N_2_) gas was then bubbled through the solution for 20 min to remove oxygen. The calculated amount of APS was added, and the temperature was raised to the polymerization temperature. Then, the calculated amount of the CTA, followed by 8 mL of acrylonitrile, was added. The reaction system was then sealed and maintained at the polymerization temperature for a specified time. Upon completion of the reaction, 50 mL of deionized water was added for rapid cooling, and the mixture was filtered via vacuum filtration. The collected product was dried in a vacuum oven at 70 °C for 24 h to obtain the desired PAN.

### 2.3. Characterization and Testing

#### 2.3.1. Characterization of Molecular Chain Structure

^1^H NMR of PAN was performed using a Bruker AV400 (Bruker Corporation, Karlsruhe, Germany) NMR machine. Deuterated dimethyl sulfoxide (DMSO-d_6_) containing an internal standard of tetramethylsilane was used as a solvent for ^1^H NMR spectral analysis. FT-IR spectra were recorded with a Perkin-Elmer Spectrum GX (PerkinElmer, Inc., MA, USA). The PAN powder was mixed with dry KBr (1:100 ratio), ground homogeneously, and pressed into transparent pellets. Measurements were performed in the range of 4000–400 cm^−1^.

#### 2.3.2. Molecular Weight and Yield Determination

GPC for characterizing M_n_ and weight average molecular weight (M_w_) and polymer dispersion index (PDI) characterization were conducted using an Agilent 1260 Infinity Ⅱ (Agilent Technologies, CA, USA) system equipped with a Waters 2414 (Waters Corporation, MA, USA) refractive index detector, using DMF as the mobile phase. The viscosity method was employed to determine the M_η_; a detailed description of this method is provided in the supporting information. The yield was calculated as the mass ratio of the acrylonitrile monomer to the dried polymerization product.

#### 2.3.3. Morphological Analysis

The surface morphology was characterized using an FE-SEM (Ultim Max40, Oxford Instruments, Oxford, UK). The PAN samples designated for FE-SEM characterization were freeze-dried under vacuum (−30 °C; 30 h) and subsequently pulverized into a fine powder before analysis.

#### 2.3.4. Thermal Performance Analysis

TGA was performed with a NETZSC STA 449F5 (NETZSCH-Gerätebau GmbH, Selb, Germany), where samples of each formulation were heated at a rate of 10 °C/min over a temperature range of 25 to 600 °C under a N_2_ flow of 20 mL/min. DSC was employed to measure the heat absorption/release reaction using a NETZSCH DSC 214 (NETZSCH-Gerätebau GmbH, Selb, Germany) under a constant N_2_ flow. The samples were heated to 280 °C at a rate of 10 °C/min. The state change was measured using a Digipol-M80 (Jiahang Instruments, Shanghai, China). The samples were heated at a heating rate of 20 °C/min until completely carbonization.

#### 2.3.5. Solubility Test

The PAN powders with distinct molecular weights were dissolved in 10 mL of the solvent (DMF/THF), subjected to 10 min of sonication, and visually compared via photographic documentation before the dissolution state analysis.

## 3. Results and Discussion

Based on aqueous precipitation polymerization, PAN with different molecular weights was obtained by adjusting different experimental conditions. The experimental parameters and corresponding molecular weight data are summarized in Table 1.

**Figure 3:**
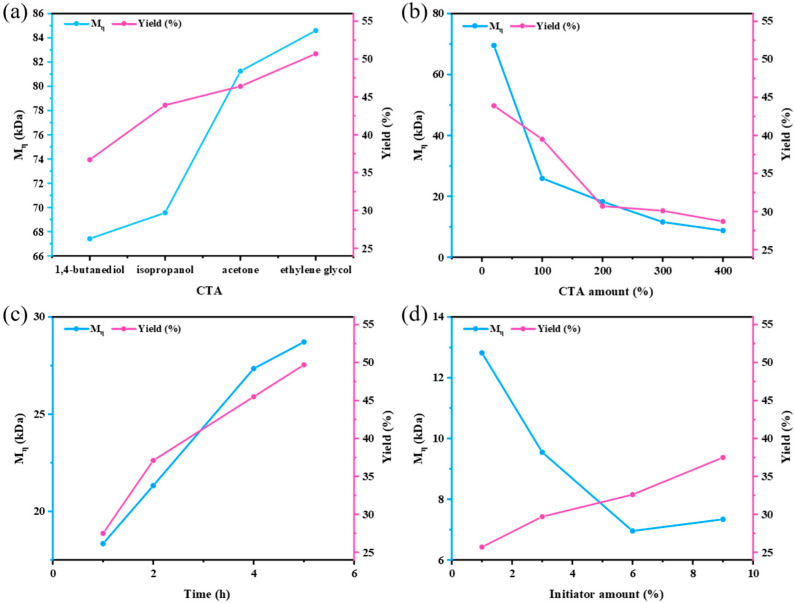
(**a**) The effect of different CTAs on M_η_. (**b**) The effect of CTA dosage relative to acrylonitrile on M_η_. (**c**) The effect of different reaction times on M_η_. (**d**) The effect of initiator addition amounts relative to acrylonitrile on M_η_.

PAN samples with different molecular weights were synthesized by introducing various green and cost-effective alcohols (i.e., ethylene glycol, IPA, and 1,4-butanediol) into the standard reaction and using acetone as a reference, as seen in Figure 3a. The results demonstrate that IPA and 1,4-butanediol significantly reduced the molecular weight of the generated PAN compared with acetone and ethylene glycol. Comparing the experimental results using ethylene glycol and 1,4-butanediol as the CTA suggests that the alcohol’s chain length plays a crucial role in chain transfer efficiency, with longer chains generally leading to improved efficiency in reducing molecular weight. It is known that low-molecular-weight polymers in aqueous precipitation polymerization are often accompanied by a lower conversion yield [36]. Consequently, using IPA and 1,4-butanediol as the CTA also led to lower monomer conversion yields. In general, IPA exhibited a significantly higher conversion yield than 1,4-butanediol (43.9% vs. 36.4%), while the resulting PANs had relatively similar M_η_ values (69.55 kDa vs. 64.42 KDa). By considering the economic factor (CNY 50/500 mL for IPA vs. CNY 85/500 mL for 1,4-butanediol in Sigma-Aldrich), IPA was regarded as a cost-effective and high-efficiency CTA for further investigation. The CTA dosage was also investigated in addition to the CTA types, as it significantly affected the molecular weight. Higher concentrations of the CTA increase the probability of effective polymer chain transfer to IPA, thereby enabling better regulation of molecular weight [1]. The IPA dosages were examined at 20%, 100%, 200%, 300%, and 400% relative to the mass of the monomer, as shown in Figure 3b. When the CTA amount added was less than 100%, increasing its concentration caused a significant decrease in molecular weight. Specifically, as the amount of IPA continued to rise, the rate of molecular weight reduction slowed, with the most noticeable change occurring at 300%. Additionally, the conversion rate declined along with the molecular weight at higher IPA concentrations. After considering both the experimental results and economic factors, 300% was the optimal dosage amount of IPA.

Subsequently, experiments with different reaction durations were conducted. As predicted by theory, both the molecular weight and conversion yield increased with longer reaction durations. Considering both molecular weight and yield comprehensively, 2 h was the optimal polymerization time (Figure 3c). Moreover, the dosage of the initiator significantly impacted the polymerization outcomes. The initiator decomposed at high temperatures to form free radicals that triggered polymerization. A higher concentration of the initiator led to more chains undergoing simultaneous polymerization, which decreased the molecular weight of individual chains. Thereby, initiator concentrations of 1%, 3%, 6%, and 9% relative to the monomer mass were tested. The results indicate that the molecular weight exhibited a decreasing trend up to an initiator concentration of 6%, while the conversion rate increased. When the initiator concentration was raised to 9%, the molecular weight slightly increased, but the yield continued to rise. Therefore, the optimal initiator concentration was determined to be 6 wt% relative to the monomer mass (Figure 3d). Based on our exploration of the experimental conditions, adding 300% IPA as the CTA and 6% APS as the initiator controlled the polymerization reaction. The polymerization reaction was carried out for 2 h to obtain a low-molecular-weight PAN. In the early literature, where isopropanol was employed for preparing low-molecular-weight poly(methyl methacrylate), the authors added 0.4 wt% initiator and 1.2 wt% chain transfer agent, yet the M_w_ could only be reduced to 300 kDa. This result demonstrates the advantages of our experimental design [29].

PAN’s properties and structure were analyzed using FE-SEM, FT-IR, and ^1^H NMR, and its thermal characteristics were evaluated using TGA, DSC, and melting point testing. To clarify the differentiation, PAN samples with four distinct molecular weights were designated as PAN-1, PAN-2, PAN-3, and PAN-4. PAN-1 was obtained under optimized experimental conditions. For comparison, PAN-2, PAN-3 (synthesized under different reaction conditions), and commercially available PAN-4 were tested. The results were subsequently compared and analyzed. The molecular weight test results for all four materials are provided in Table 2.

**Figure 4:**
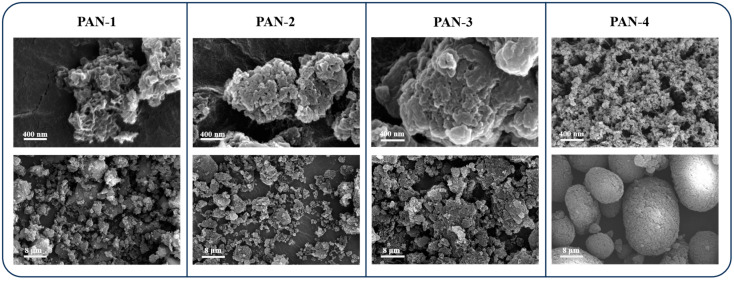
SEM images of PAN-1, PAN-2, PAN-3, and PAN-4 at different scales.

The morphological characterization of the four samples using FE-SEM revealed that the particle sizes obtained via aqueous precipitation polymerization were non-uniform, as shown in Figure 4. The low-molecular-weight PAN samples, PAN-1 (6.8 kDa) and PAN-2 (11.56 kDa), consisted of numerous submicron-sized particles. Further comparing these two polymers showed that when the molecular weight difference was within 10 kDa, it was difficult to discern variations in molecular weight from SEM images alone. The synthesized PAN-3 with a higher molecular weight (69.6 kDa) exhibited a similar morphology to that of the low-molecular-weight PAN-1 and PAN-2. However, its particles were composed of micrometer-sized aggregates. In contrast, the commercially available PAN-4 (M_n_: 105.3 kDa) displayed a more regular shape, a smoother surface, and a slightly larger particle size than PAN-3. The PAN synthesized in this study featured a smaller particle size but exhibited a rougher surface and some particle agglomeration.

**Figure 5:**
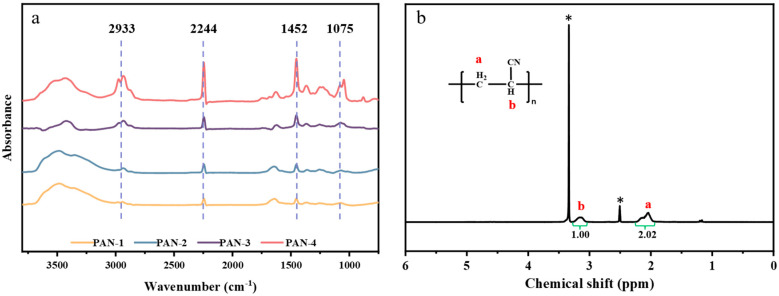
(**a**) FT-IR spectra of PAN-1, PAN-2, PAN-3, and PAN-4. (**b**) ^1^H NMR spectrum of PAN-1. The spectrum showed two additional signal peaks at δ 3.3 ppm and δ 2.5 ppm (*), which were primarily due to the presence of water and residual protons from deuterated DMSO-d₆, respectively [39].

FT-IR was employed to characterize the structures of PAN-1, PAN-2, PAN-3, and PAN-4, recording the spectra in the wavenumber range of 4000–400 cm^−1^ (Figure 5a). An analysis of the results revealed that the peaks at 1452 cm^−1^ and 2933 cm^−1^ were attributed to C-H bond in-plane bending vibration and stretching vibration, respectively [37]. A distinct sharp peak at 2244 cm^−1^ corresponded to the C≡N bond stretching vibration unique to cyano groups, while the absorption peak at 1075 cm^−1^ represented the stretching vibration of the C-CN bond. Thus, the spectral features confirmed that the product was a homopolymer structure of PAN [38]. The structure of the target low-molecular-weight PAN-1 was further characterized using ^1^H NMR. As shown in Figure 5b, the ^1^H NMR spectrum of PAN-1 revealed two prominent signal peaks at chemical shifts of δ 2.04 ppm and δ 3.15 ppm, which corresponded to hydrogen atoms with different chemical shifts, denoted as peaks a and b, respectively. Peak a was assigned to the -CH₂ hydrogen atoms, while peak b corresponded to the -CH hydrogen atoms in the PAN chain [37]. These results confirm that the product obtained via aqueous precipitation polymerization was PAN. The spectrum showed two additional signal peaks at δ 3.3 ppm and δ 2.5 ppm, which were primarily due to the presence of water and residual protons from deuterated DMSO-d₆, respectively [39].

**Figure 6:**
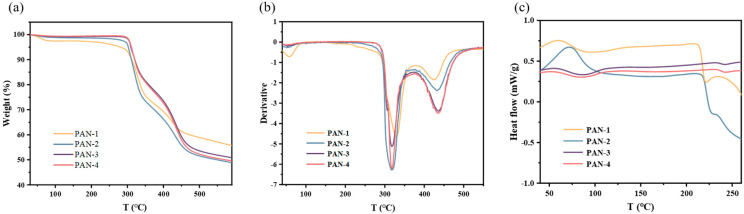
(**a**) TGA curves of PAN-1, PAN-2, PAN-3, and PAN-4 at a heating rate of 10 °C/min in N_2_. (**b**) Derivative of TGA curves for PAN-1, PAN-2, PAN-3, and PAN-4. (**c**) DSC curves of PAN-1, PAN-2, PAN-3, and PAN-4 at a heating rate of 10 °C/min in N_2_.

For decades, PAN has served as a crucial precursor for producing carbon fibers; as such, its thermal degradation constantly attracts considerable attention [40]. Understanding the thermal oxidation stability process is essential for studying the high-temperature carbonization of PAN [41,42]. It is widely recognized that the chemical reactions occurring during the thermal degradation of PAN primarily involve cyclization, dehydrogenation, and oxidation [43]. While cyclization and dehydrogenation are regarded as the dominant changes in the initial stabilization phase, the precise sequence and mechanisms of these reactions remained unclear [44,45]. Nevertheless, most of these chemical transformations are accompanied by changes in appearance or weight [46]. PAN samples with varying molecular weights were subjected to TGA, DSC, and melting point analysis to investigate the intriguing relationship between molecular weight and thermal properties. Figure 6a illustrates the thermogravimetric curves for PAN-1, PAN-2, PAN-3, and PAN-4 in an N_2_ atmosphere at a heating rate of 10 °C/min. The TGA results reveal that all four materials exhibited a similar weight loss pattern, initiating at around 240 °C and stabilizing at approximately 550 °C, with a mass loss of approximately 50%. PAN-1, which had the lowest molecular weight, began to lose weight first, while the higher-molecular-weight samples showed a delayed onset of weight loss. These results are consistent with theoretical predictions, indicating that shorter polymer chains lead to reduced interchain entanglement and an increased proportion of chain ends. On the one hand, this weakens intermolecular forces, making the material more prone to chain dynamics and softening upon heating. On the other hand, the abundant chain ends act as the initiation sites for thermal decomposition [47,48]. Figure 6b presents the derivative of the TGA curve, which further emphasizes that PAN with a lower molecular weight experienced weight loss earlier. Additionally, the TGA curve indicates that all four materials exhibited two significant peak temperatures at 318 °C and 433 °C during weight loss, which can be attributed to HCN release and NH₃ release, respectively [5]. Figure 6c displays the DSC analysis of the four PAN samples under N_2_ at a heating rate of 10 °C/min over a temperature range of 25 °C to 280 °C. Consistent with the TGA results, the DSC data further support the conclusion that lower-molecular-weight PAN undergoes intramolecular reactions more readily as the temperature increases. This highlights the significance of low-molecular-weight PAN in producing tetrazolium compounds, as it possesses higher chemical reactivity, making the cyano group more prone to [3 + 2] addition reactions with azide groups [5].

**Figure 7:**
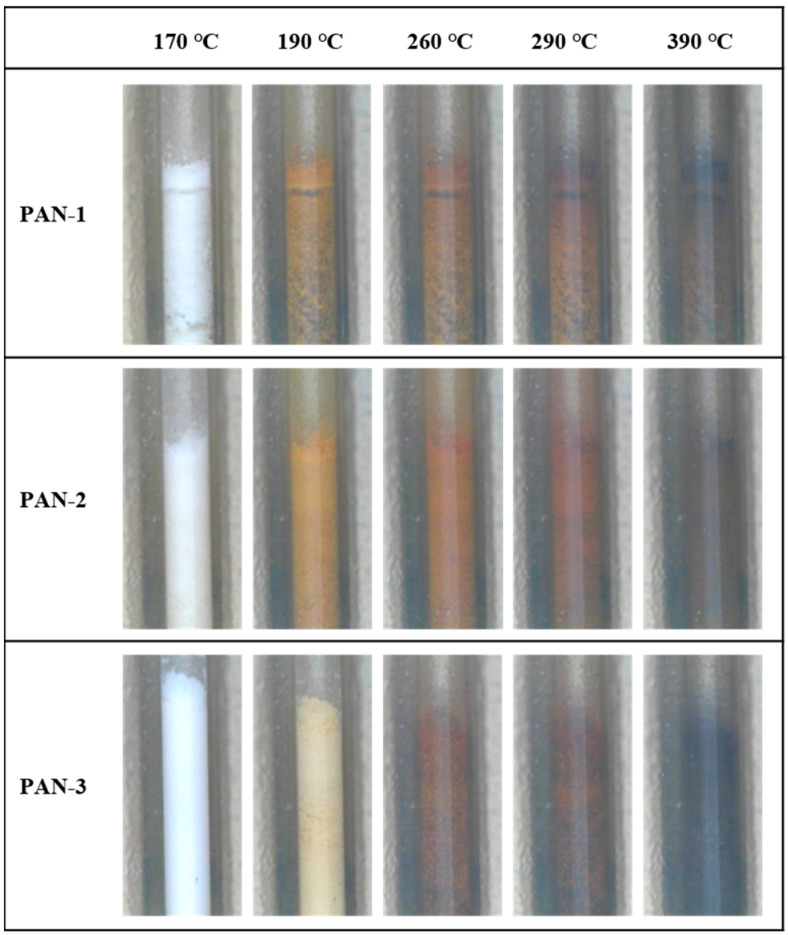
Variation diagram of PAN-1, PAN-2, and PAN-3 at different temperatures in melting point analyzer.

The thermal property characterization of PAN using TGA or DSC relies on plotting data recorded with instruments and analyzing key information [49]. However, these measurements cannot provide a direct observation of the state change in PAN with temperature increases. To address the issue, a visual melting point analyzer was applied to the synthesized PAN-1, PAN-2, and PAN-3 samples (Figure 7). The heating process was performed in a N_2_ atmosphere with a temperature ranging from room temperature to 390 °C at a heating rate of 20 °C/min. In general, obvious color and state changes of PAN with different molecular weights were observed with an elevation in temperature. All three samples began to phase change in the temperature range between 170 °C and 190 °C. This change could be attributed to the partial oxidation of PAN and the cyclization of the polymer chains. PAN-1, with the lowest molecular weight, exhibited the most rapid color change, aligning with the abovementioned results. As the temperature continued to rise, all three materials gradually carbonized, likely due to accelerated cyclization and dehydrogenation reactions, which increased the overall carbon content of the materials [50]. Although the theoretical melting point of PAN is approximately 317 °C, cyclization and dehydrogenation begin at 230 °C in an air environment, resulting in significant changes to the material [51]. Consequently, no melting behavior was observed in the melting point analyzer.

**Figure 8:**
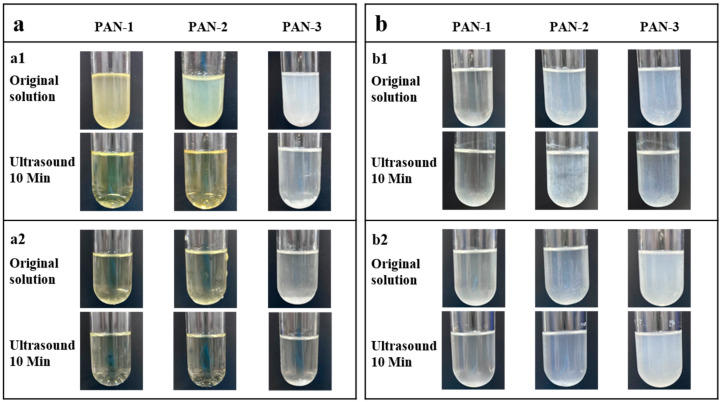
(**a**) Solubility comparison of PAN-1, PAN-2, and PAN-3 in DMF at different concentrations: (**a1**) 20 mg/mL and (**a2**) 10 mg/mL. (**b**) Solubility comparison of PAN-1, PAN-2, and PAN-3 in THF and IPA: (**b1**) 2 mg/mL in THF; (**b2**) 2 mg/mL in IPA.

Solubility, another important physical property for polymer materials, impacts fundamental research as well as industrial applications [52,53]. For example, the solubility of PAN significantly influences its processing techniques, such as injection molding, spraying, and pouring [54]. Selecting and optimizing appropriate solvents in the experimental process can enhance processing efficiency, minimize production waste, reduce costs, and improve product quality [55]. Therefore, the solubilities of PAN-1, PAN-2, and PAN-3 were investigated in detail. The samples were first dissolved in DMF, and solutions were prepared at concentrations of 20 mg/mL and 10 mg/mL. Their appearances are illustrated in Figure 8(a1) and Figure 8(a2), respectively. Then, the solutions underwent ultrasonication for 10 min, and their appearances after treatment were recorded for comparison. The results indicate that low-molecular-weight PAN exhibited good solubility in DMF. Upon comparing the three polymers, the low-molecular-weight PAN-1 solution appeared light yellow, likely due to its poor heat resistance and degradation during the drying process. The color of the solution gradually lightened as the molecular weight increased, corroborating our previous findings on PAN’s thermal properties. A comparison of the solution appearance at a concentration of 20 mg/mL demonstrated that PAN with lower molecular weights possessed higher solubilities than PAN with higher molecular weights, as the solutions of PAN-1 and PAN-2 were more transparent than that of PAN-3 (Figure 8a). Additionally, the solubilities of PAN in THF (Figure 8(b1)) and IPA (Figure 8(b2)) were examined. The poor solubility of PAN in both solvents limited the dispersion concentration to 2 mg/mL for comparison. Although PAN showed slightly better solubility in IPA than in THF, the solubility remained relatively low in both solvents, and no significant improvement was observed even after ultrasonic treatment. Nevertheless, similar to the DMF experiment, PAN’s solubility in IPA and THF also decreased with increasing molecular weight.

**Figure 9:**
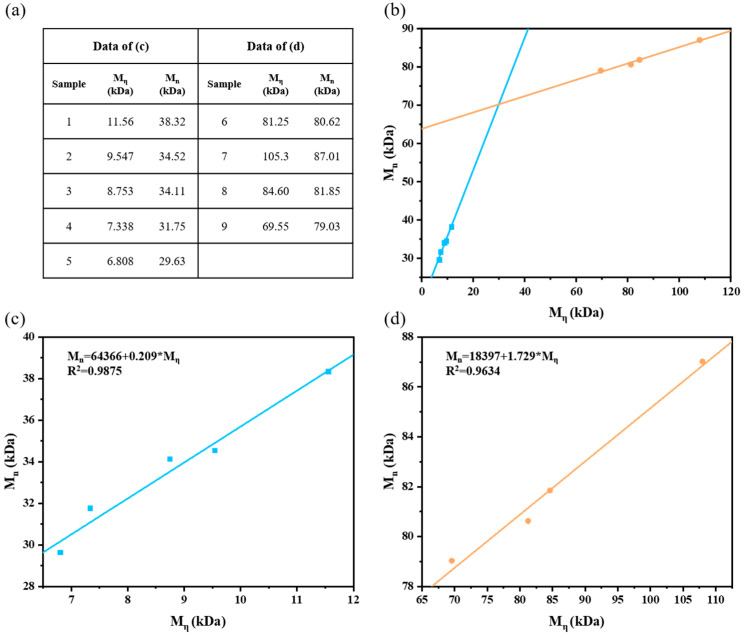
(**a**) Summary table of PAN with different molecular weights. (**b**) Fit plot of M_η_ and M_n_ of PAN with different molecular weights. (**c**) Fit plot of M_η_ and M_n_ of low-molecular-weight PAN. (**d**) Fit plot of M_η_ and M_n_ of high-molecular-weight PAN.

Polymers are characterized primarily using structural and molecular weight analysis, and GPC is the main method for measuring their molecular weight [56]. However, GPC suffers from issues such as being time-consuming, complex to operate, and costly, which pose challenges in the process design of polymer materials that often require extensive experimentation and multiple sample analyses. Given these constraints, efficient and cost-effective methods to assess polymer molecular weights using laboratory settings are urgently needed. One simple technique for this purpose is the M_η_ measurement, which uses an Ubbelohde viscometer to measure the viscosity of polymer solutions and calculate the M_η_ values using the formulas. Details of the experimental procedure are provided in the Supporting Materials. Although both the GPC and viscosity methods have inherent errors when characterizing low-molecular-weight polymers, GPC results are generally considered more reliable. This study employed the GPC and viscosity methods to jointly analyze the molecular weights of nine representative PAN samples with varying molecular weights and compared the relationship between M_n_ and M_η_ via linear regression (Figure 9a). The results revealed a strong linear regression between M_n_ and M_η_ (Figure 9b). Furthermore, two distinct linear functions were obtained in different molecular weight ranges, with an intersection at an M_η_ of 29.9 kDa (for details of the two lines, please refer to Figure 9c,d). This point represents a crucial threshold: when the molecular weight reaches 29.9 kDa, the high molecular weight characteristic should be applied; conversely, the low molecular weight function should be used when M_η_ is below this value. Our findings provide valuable insights for evaluating polymer molecular weights in laboratory settings and may enhance the efficiency of polymer material process designs.

## 4. Conclusions

In this study, aqueous precipitation polymerization with a CTA was developed to regulate the polymerization degree of PAN, resulting in the synthesis of low-molecular-weight PAN. During the synthetic process, the effects of different CTAs on the polymerization outcomes were studied. CTAs with longer chain lengths were found beneficial for synthesizing lower-molecular-weight PAN. Low-molecular-weight PAN (M_η_: 6.808 kDa) was successfully synthesized via polymerization at 70 °C for 2 h under optimized experimental conditions, employing APS (6 wt% relative to the monomer mass) as the initiator and IPA (400 wt%) as the CTA. PAN samples with different molecular weights were successfully obtained via extensive parallel experiments. FE-SEM observation showed that the synthesized PAN surface was relatively rough, and the particle size was smaller than that of high-molecular-weight PAN. The structure of the synthesized PAN was confirmed using FT-IR and ^1^H NMR. Additionally, the relationship between molecular weight and both thermal properties and solubility was systematically investigated. Thermal analysis via TGA and DSC indicated that lower-molecular-weight PAN exhibited higher reactivity and a lower decomposition temperature. Therefore, PAN with the lowest molecular weight was observed to undergo oxidation and turn yellow first, as detected with a visual melting point analyzer. Since solubility is a critical property of polymers, the solubilities of PAN in DMF, IPA, and THF were assessed, finding that DMF provided the best solubility. Our results further confirmed that lower-molecular-weight PAN demonstrated enhanced solubility. Finally, a strong linear regression was established between M_η_ and M_n_, which offered a rapid and simple polymer molecular weight measurement using laboratory settings.

## Figures and Tables

**Table 1:** Process formula and molecular weight measurement results of PAN.

Sample	CTA	AN–CTA–APS	Time (h)	M_η_ (kDa)	M_n_ (kDa)	M_w_ (kDa)	PDI	Yield (wt%)
1	Acetone	100:20:3	3	81.25	80.62	223.9	2.78	46.4
2	Glycol	100:20:3	3	84.60	81.85	229.45	2.81	50.7
PAN-3	Isopropanol	100:20:3	3	69.55	79.03	207.79	2.63	43.9
4	1,4-butanediol	100:20:3	3	64.42	/	/	/	36.4
5	Isopropanol	100:100:3	3	25.90	/	/	/	39.5
6	Isopropanol	100:200:3	3	18.26	/	/	/	30.7
PAN-2	Isopropanol	100:300:3	3	11.56	38.32	62.40	1.63	30.3
8	Isopropanol	100:400:3	3	8.753	34.11	64.84	1.91	28.3
9	Isopropanol	100:100:3	1	18.34	/	/	/	27.5
10	Isopropanol	100:100:3	2	21.33	/	/	/	37.1
11	Isopropanol	100:100:3	4	27.35	/	/	/	45.5
12	Isopropanol	100:100:3	5	28.71	/	/	/	49.8
13	Isopropanol	100:300:1	2	12.82				25.2
14	Isopropanol	100:300:3	2	9.547	34.52	59.53	1.72	29.7
PAN-1	Isopropanol	100:300:6	2	6.808	29.63	58.50	1.97	32.6
16	Isopropanol	100:300:9	2	7.338	31.75	51.47	1.62	37.5

**Table 2:** The molecular weights of PAN-1, PAN-2, PAN-3, and PAN-4. M_n_, M_w_, and PDI measured via GPC; M_η_ measured using viscosity method. The yield is the mass ratio of acrylonitrile to the polymerization product.

Sample	M_η_ (kDa)	M_n_ (kDa)	M_w_ (kDa)	PDI	Yield (wt%)
PAN-1	6.808	29.63	58.50	1.97	32.6
PAN-2	11.56	38.32	62.40	1.63	30.3
PAN-3	69.55	79.03	207.8	2.63	43.9
PAN-4	105.3	87.01	238.1	2.74	/

## Data Availability

The original contributions presented in this study are included in the article/supplementary material. Further inquiries can be directed to the corresponding authors.

## Supplementary Materials

The following supporting information can be downloaded at https://www.mdpi.com/article/10.3390/polym17081112/s1. Figure S1: Structure of the Ubbelohde viscometer; Table S1: Data integration of viscosity method for testing molecular weight; Figure S2: Extrapolation method for calculating intrinsic viscosity [η]; Figure S3: Molecular weight distributions of PAN-1, PAN-2, PAN-3, and PAN-4 determined using GPC; Figure S4: Molecular weight distributions of other tested samples determined using GPC. References [57,58,59,60,61] are cited in the supplementary materials.
