# Supplementary material for: Preparation and Characterization of Low-Molecular-Weight Polyacrylonitrile
Source: Polymers (Basel). 2025 Apr 19;17(8):1112. doi: 10.3390/polym17081112 (PMC12030532; doi:10.3390/polym17081112)
Supplement: Supplementary file 1 [file polymers-17-01112-s001.zip › polymers-3594488-supplementary.pdf]

Supporting Information

Preparation and Characterization of Low-Molecular-Weight Polyacrylonitrile

Viscosity method for testing viscosity average molecular weight: The sample material was dissolved in DMF and a solution of 10 mg mL^{-1} was prepared. The Ubbelohde viscometer was placed in a water bath and the temperature was maintained at 25°C for 10 minutes. Then 10 mL of the prepared solution was added to the Ubbelohde viscometer from Filling tube, and the system was left in a water bath for 10 minutes. Then seal venting tube and use a rubber suction bulb to suck the liquid level from capillary tube to the feeder bulb (Fig. S1). Open venting tube and start timing when the liquid level drops to the M1. Stop timing when the liquid level drops to the M2. Repeat this process three times with a time error less than 0.3 seconds and record the average time value t at a concentration of C1. Subsequently, 5 mL of DMF was added to the viscometer and the solution was diluted evenly to a concentration of C2, Use the above method to test the time at C2. Further test the time of C3 and C4. Finally, place 20 mL of solvent DMF in a viscometer to measure the viscosity of the solvent, and test for time t0 using the same method as above [1].

Formula calculation: $\eta_{sp}=(\eta-\eta_0)/\eta_0=(t-t_0)/t_0$, η_{sp}: Increasing specific viscosity

$\eta_r=\eta/\eta_0=t/t_0$ η_r: Specific viscosity

Calculate all data using formulas, as the example of Table S1. Plot the concentration c of η_{sp}/c and $\ln\eta_r/c$, as shown in Fig. S2 [2,3]. Linearly fit the two sets of data, and they will intersect at a point on the y-axis, which is the intrinsic viscosity [η] of PAN. According to the Mark-Houwink Equation $[\eta]=2.33 \times 10^{-4}M_\eta^{0.75}$, the viscosity average molecular weight (M_η) can be obtained [4,5].

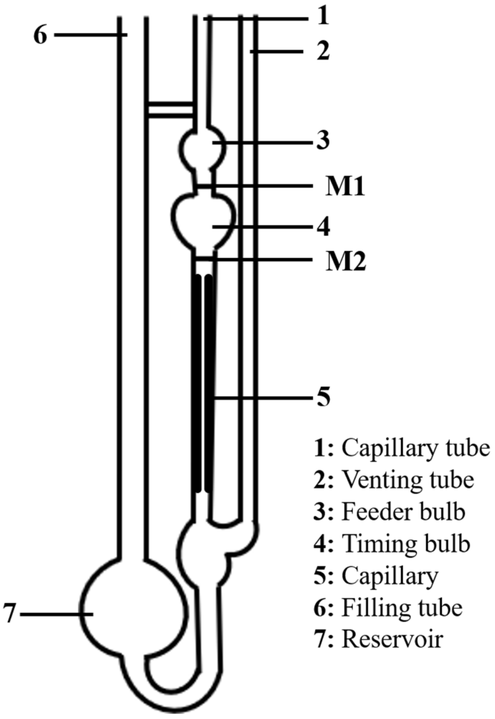

Figure S1. Structure of the Ubbelohde viscometer.

Table S1. Data integration of viscosity method for testing molecular weight.

	Concentration/(mg·L^{-1})	Pure solven t_0	Solvent t	$\eta_r=t/t_0$	$\ln\eta_r/c$	$\eta_{sp}=\eta_r-1$	η_{sp}/c
c1	10	108.26	127.8	1.18213	0.01673	0.01821	0.01821
c2	6.7	108.26	121.1	1.12016	0.01701	0.01201	0.01801
c3	5.0	108.26	117.9	1.08945	0.01713	0.08945	0.01788
c4	4.0	108.26	115.8	1.07113	0.01717	0.07113	0.01778

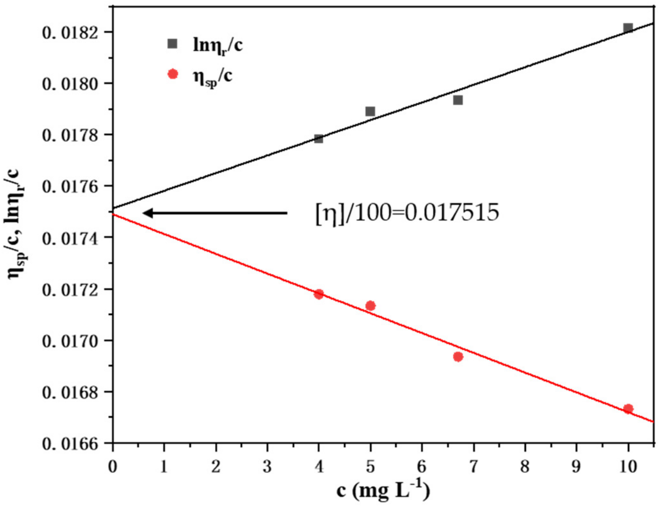

Figure S2. Extrapolation method for calculating intrinsic viscosity [η].

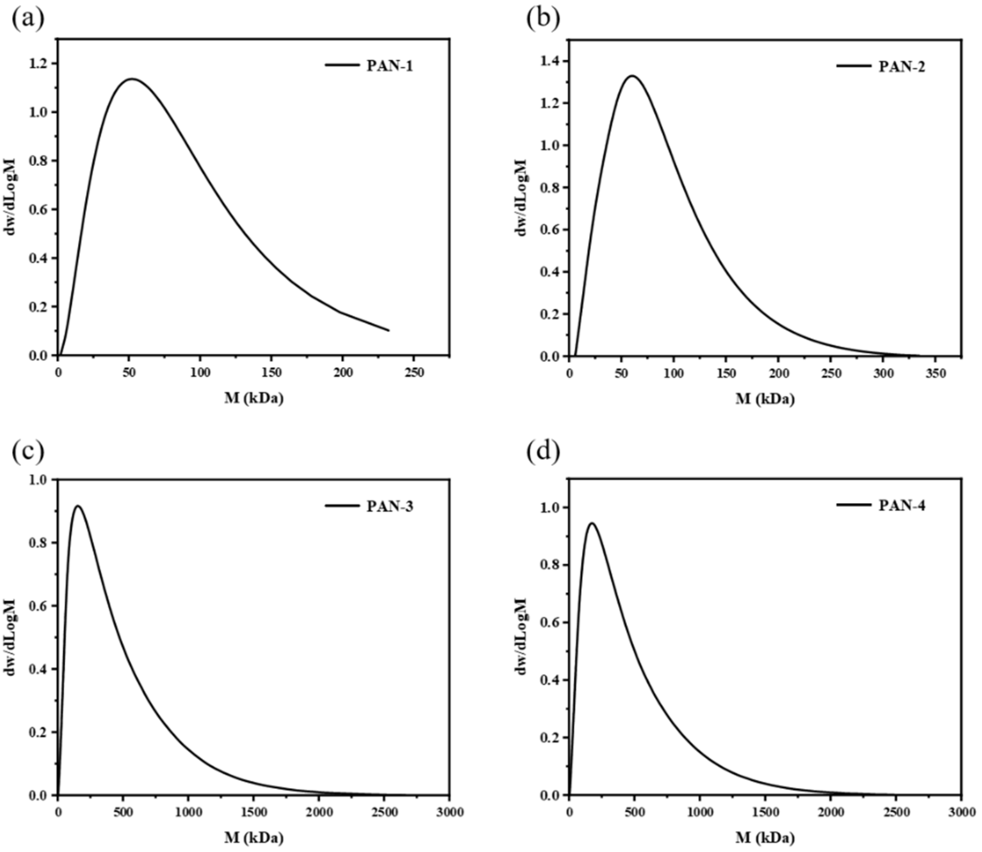

Figure S3. Molecular weight distributions of PAN-1, PAN-2, PAN-3 and PAN-4 determined by GPC.

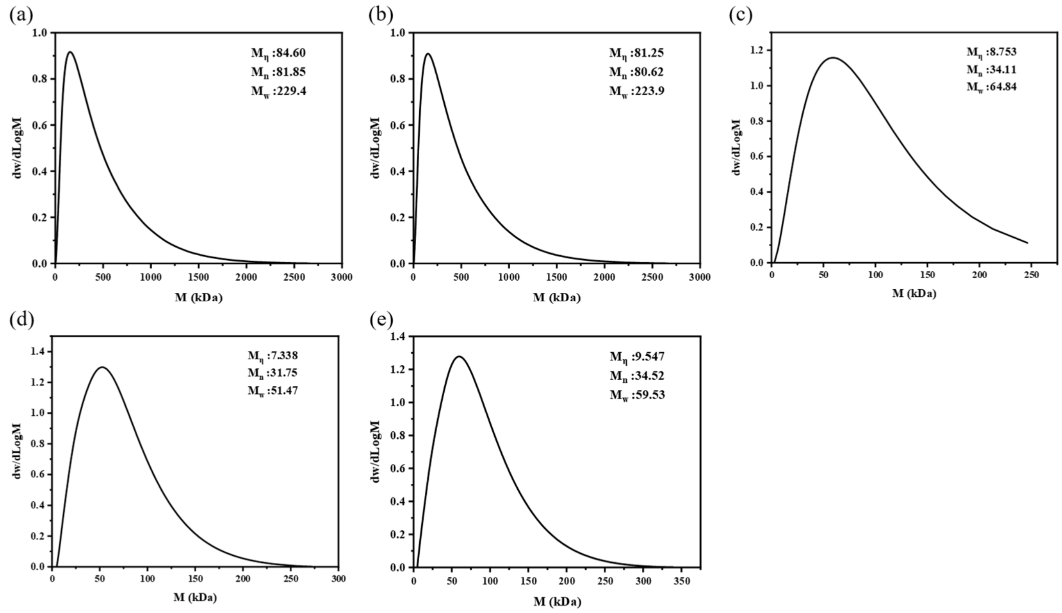

Figure S4. Molecular weight distributions of other tested samples determined by GPC.

References

1. Bos, T.S.; Philipsen, H.J.; Staal, B.B.; Purmova, J.; Beerends, R.J.; Buijtenhuijs, A.; Karlson, L.; Schoenmakers, P.J.; Somsen, G.W. Quantitative assessment of polymer molecular shape based on changes in the slope of the Mark-Houwink plot derived from size-exclusion chromatography with triple detection. *Journal of Applied Polymer Science* **2024**, *141*, e55013, doi: 10.1002/app.55013
2. Al-Dujaili, A.H.; Mustafa, I.F. Viscometric behavior of solution of polyisobutylene in hexane and cyclohexane solvents: viscosity-molecular weight relationships. *Polymer-Plastics Technology and Engineering* **1994**, *33*, 111-118, doi: 10.1080/03602559408010735
3. Chuah, H.; Lin-Vien, D.; Soni, U. Poly (trimethylene terephthalate) molecular weight and Mark–Houwink equation. *Polymer* **2001**, *42*, 7137-7139, doi: 10.1016/S0032-3861(01)00043-X
4. Holdcroft, S. Determination of molecular weights and Mark–Houwink constants for soluble electronically conducting polymers. *Journal of Polymer Science Part B: Polymer Physics* **1991**, *29*, 1585-1588, doi: 10.1002/polb.1991.090291303
5. Schott, H. Dependence of the Constant of the Mark-Houwink Equation under Theta Conditions on the Bulk of Substituents for Hydrocarbon Polymers. *Journal of Macromolecular Science, Part B: Physics* **2006**, *45*, 1183-1187 , doi: 10.1080/00222340600929717
